# Midbrain hypoactivation and mesocortical hypoconnectivity in inhibitory control learning in autism

**DOI:** 10.1093/braincomms/fcag265

**Published:** 2026-07-12

**Authors:** Ana Araújo, Isabel C Duarte, Teresa Sousa, Graça Areias, Ana T Pereira, Trevor W Robbins, António Macedo, Miguel Castelo-Branco

**Affiliations:** Coimbra Institute for Biomedical Imaging and Translational Research (CIBIT), University of Coimbra, Coimbra 3000-548, Portugal; Institute for Nuclear Sciences Applied to Health (ICNAS), University of Coimbra, Coimbra 3000-548, Portugal; Institute of Psychological Medicine, Faculty of Medicine, University of Coimbra, Coimbra 3004-504, Portugal; Department of Psychiatry, Local Health Unit of Coimbra, Coimbra 3004-561, Portugal; Coimbra Institute for Biomedical Imaging and Translational Research (CIBIT), University of Coimbra, Coimbra 3000-548, Portugal; Institute for Nuclear Sciences Applied to Health (ICNAS), University of Coimbra, Coimbra 3000-548, Portugal; Coimbra Institute for Biomedical Imaging and Translational Research (CIBIT), University of Coimbra, Coimbra 3000-548, Portugal; Institute for Nuclear Sciences Applied to Health (ICNAS), University of Coimbra, Coimbra 3000-548, Portugal; Institute of Physiology, Faculty of Medicine, University of Coimbra, Coimbra 3004-531, Portugal; Department of Psychology, Local Health Unit of Coimbra, Coimbra 3004-561, Portugal; Coimbra Institute for Biomedical Imaging and Translational Research (CIBIT), University of Coimbra, Coimbra 3000-548, Portugal; Institute for Nuclear Sciences Applied to Health (ICNAS), University of Coimbra, Coimbra 3000-548, Portugal; Institute of Psychological Medicine, Faculty of Medicine, University of Coimbra, Coimbra 3004-504, Portugal; Department of Psychology, Behavioral and Clinical Neuroscience Institute, University of Cambridge, Cambridge CB2 3EB, UK; Coimbra Institute for Biomedical Imaging and Translational Research (CIBIT), University of Coimbra, Coimbra 3000-548, Portugal; Institute for Nuclear Sciences Applied to Health (ICNAS), University of Coimbra, Coimbra 3000-548, Portugal; Institute of Psychological Medicine, Faculty of Medicine, University of Coimbra, Coimbra 3004-504, Portugal; Department of Psychiatry, Local Health Unit of Coimbra, Coimbra 3004-561, Portugal; Coimbra Institute for Biomedical Imaging and Translational Research (CIBIT), University of Coimbra, Coimbra 3000-548, Portugal; Institute for Nuclear Sciences Applied to Health (ICNAS), University of Coimbra, Coimbra 3000-548, Portugal; Institute of Physiology, Faculty of Medicine, University of Coimbra, Coimbra 3004-531, Portugal

**Keywords:** striatum, midbrain, stop-signal task, error valuation, dopamine

## Abstract

Repetitive behaviour, resulting from impaired inhibitory control and error monitoring, represents a core manifestation of autism, with a poorly understood neural basis. Our primary hypothesis was that a striatum-midbrain framework could provide a theoretical basis for understanding neural changes underlying response inhibition in autism. This conceptual approach considered two critical neurobehavioural factors: (a) inhibition as a dynamic process requiring trial-and-error learning, and (b) efficient error learning relying on the striatum receiving dopaminergic signals from the midbrain—a framework analogous to an ‘actor-critic’ architecture. Eighteen adults with a diagnosis of autism spectrum disorder and 21 age-matched healthy controls performed a stop-signal task adjusted for functional MRI (fMRI). To dissect domain-dependent correlates of neural inhibition, we measured brain activation and connectivity as a function of task phases in the dorsal striatum and dopaminergic midbrain nuclei. Repetitive behaviour severity was assessed using the observer-reported Repetitive Behaviours Scale—Revised. A striking hypoactivation in the midbrain during failed inhibition events was observed in relation with the severity of repetitive behaviours. We also identified, in the autism group, reduced functional connectivity between the midbrain-striatum hubs and regions involved in cognitive control (prefrontal cortex) and error monitoring (bilateral insula), during response preparation periods. Finally, although both groups achieved similar final performance levels, neurodivergent subjects learned slower and displayed delayed striatal engagement when behavioural adjustment was required. These results reveal a novel autism profile characterized by midbrain hypoactivation mediating repetitive behaviour manifestations and reduced long-range mesocortical hypoconnectivity during inhibitory response preparation phases. Accordingly, we suggest that individuals with autism exhibit midbrain-dependent reduced motivational arousal, limiting their ability to develop proactive strategies for trial-and-error learning and regulate out-of-context behaviours. By highlighting the key role of dopaminergic midbrain structures and related long-range pathways, we challenge the view autism as solely a cortical dysfunction condition and provide evidence for promising targets for neurobiologically-driven interventions based on dopaminergic mechanisms.

## Teaser

Araujo *et al*. show domain and timing-dependent midbrain hypoactivation and mesocortical hypoconnectivity in adult males with autism during error-related and proactive phases of inhibitory learning. These results highlight a key role of dopaminergic structures in motivational arousal and learning styles of individuals with autism, unveiling potentially new treatment targets.

## Graphical Abstract

**Graphical Abstract:**
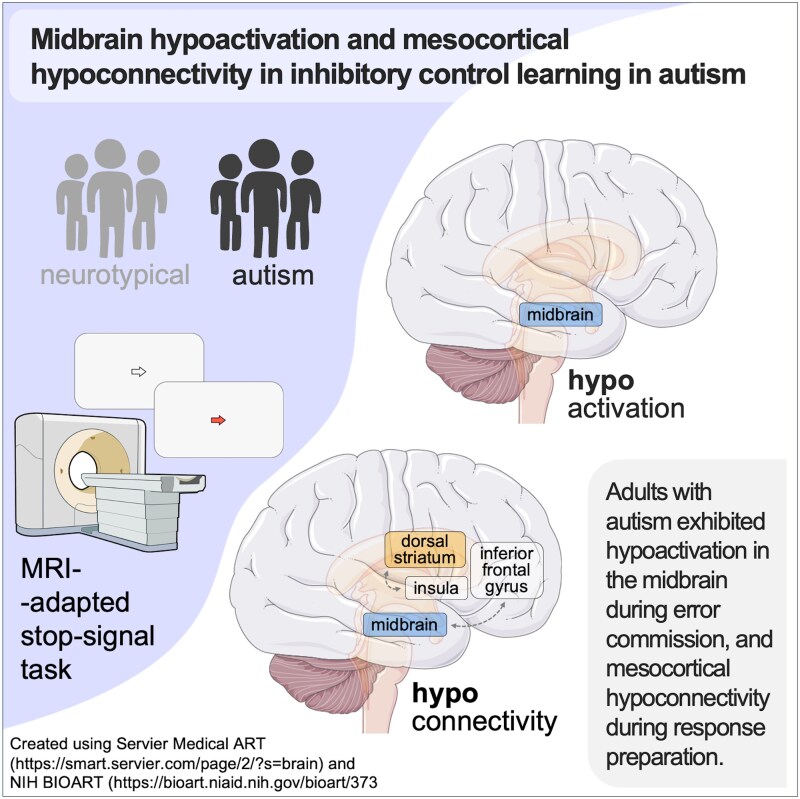

## Introduction

Individuals within the autism spectrum exhibit divergent information processing modes.^1,2^ This notion of neurodiversity calls for a paradigm shift away from an exclusive focus on the deficits to the differences associated with autism. Advocacy groups, family organizations, and clinicians are now converging to the need to identify the explanatory routes leading to diverse neural processing styles and, sometimes, to functional disability. Similarly, advancing our neurobiological understanding of the autistic brain depends on the adoption of realistic experimental approaches that can integrate multilevel findings into coherent and comprehensive frameworks.^3-5^ A prominent view based on underconnectivity^6,7^ suggests that individuals with autism, due to large-scale neural network desynchronization, use alternative cognitive strategies. This theory posits that the neurodevelopmental trajectories in autism fail to produce the typical overtaking of short-range connections by long-range connections, thereby impairing the development of core functions such as control of response inhibition.^8^

Altered response inhibition is thought to underlie the expression of repetitive behaviours (RB), which constitute core diagnostic features of autism.^2,8-12^ Neuroimaging evidence points to the involvement of brain regions within the inhibitory control network, such as the basal ganglia, inferior frontal gyrus, anterior cingulate cortex, and premotor areas.^2,8-12^ However, the nature of such changes remains controversial. For instance, different studies have reported varying inhibition-related activity levels in the striatum of individuals with autism—ranging from increased,^10^ decreased,^12^ or unchanged^8^—when compared with neurotypical subjects. Additionally, intact behavioural performance in some inhibition tasks has been identified in this condition.^5,8^

The existing literature discrepancies regarding alterations of inhibition in autism notably contrast with the scarcity of procedures for integrating objective data from neuroimaging and neurobehavioural measures into explanations of complex human cognitive processes.^13^ We tried to overcome these limitations by examining inhibition as an ecological construct inherently susceptible to many failures, requiring coordination with other neurocognitive functions such as trial-and-error learning.^14-16^ Although this perspective has been infrequent in research, where operationalization of multifaceted constructs into single indexes is the common practice, some studies suggest that particular subdomains, such as error monitoring,^17-21^ proactive control, and goal-directed learning,^2,8,22^ are specifically affected in autism.^23,24^ Our main goal was to focus on the dynamics and interrelatedness embedded in a set of inhibitory processes. Therefore, we developed a novel fMRI protocol based on the actor-critic framework and settled on the stop-signal task (SST)^25-27^ as a suitable approach for investigating the neural changes associated with response inhibition in autism.

The actor-critic model^28,29^ captures the fundamental mechanisms involved in trial-and-error learning, which is the type of learning essential for most human behaviours. Accordingly, an ‘actor’ module learns a policy and selects actions, while a ‘critic’ module estimates the reward value of such actions and generates a prediction error, that ‘trains’ the organism to choose the most appropriate options.^28,29^ Over time, this process contributes to the development of a reward-based learned strategy that is necessary for adaptive goal-directed behaviour.^22,30^

Driven by analogies between the prediction error and the firing pattern of dopamine neurons first identified in monkeys by Wolfram Schultz,^31,32^ our previous study in healthy humans^33^ confirmed the engagement of neural circuits linking the dorsal parts of the caudate and putamen with the lateral ventral tegmental area (VTA) and substantia nigra (SN) during the commission of inhibition errors, where these regions exhibited a neurobehavioural profile matching with the ‘actor’ and ‘critic’ roles. This framework also helped to put in perspective our observations in individuals with obsessive-compulsive disorder, where we identified an ‘actor-critic’ dichotomy marked by hypoconnectivity and hyperactivity mediating symptom severity.^34^ Here we focused on autism to test the utility of our actor-critic-based framework for isolating precise mechanisms underlying multidimensional symptoms of RBs.

The SST^25-27^ is a classic paradigm used to assess response inhibition, specifically the termination of an already initiated action (*i.e.* action cancellation). Participants are instructed to respond to go cues by pressing a button but to withhold their response when a stop cue appears after the go cue. Given that errors are inevitable, the SST inherently involves error monitoring and learning through repeated successful and failed inhibitory events, *i.e.* trial-and-error learning.^25,35,36^ Here, we defined inhibition errors as instances where the subject should have withheld their response but inadvertently pressed the button. The task algorithm is adaptive, maintaining each participant’s performance around a 50% error level,^26^ and participants are informed of this. They are also instructed that both responding quickly to go cues and successfully inhibiting button presses to stop cues are equally important, emphasizing the need to find (learn) the optimal balance between rapid response execution and accurate inhibition, referred to as the ‘speed-accuracy trade-off’.^26^

We hypothesized that adults with autism, compared with neurotypical controls, would exhibit distinct inhibition learning strategies, mediated by functional activity and connectivity alterations in the midbrain and basal ganglia, as core components of the ‘actor-critic’ model. We anticipated identifying changes during specific phases that support error-related motivational responses and proactive control mechanisms. Furthermore, consistent with findings of network desynchronization and atypical processing styles in autism, we expected to observe disruption in long-range connectivity. Lastly, we aimed to evaluate whether the actor-critic framework could elucidate the complex and multilevel relationships between neural functioning and autistic symptoms of RBs.

## Materials and methods

### Participants

The study involved 39 right-handed adult males, distributed across two age-matched groups—neurotypical subjects (*n* = 21) and individuals with autism (*n* = 18)—were included in the reported analysis. To ensure a more homogeneous sample and prevent potential confounding variables related to sex-specific neural patterns in SST activation,^37^ female participants were not included. Participant profiles and descriptive statistics are documented in Supplementary Table 1. The sample presented a *mean age* ± *standard deviation* of 27.79 ± 8.6 years, enabling the assessment of a developmental window in which inhibitory abilities are functionally mature.^37^

Individuals from the autism group were enrolled through the Unit of Adult Neurodevelopment Disorders, at the Department of Psychiatry of the Local Health Unit of Coimbra. Following the Unit’s standard clinical protocols, an experienced psychiatrist performed the diagnosis of autism spectrum disorder (ASD) based on the Diagnostic and Statistical Manual of Mental Disorders, 5th edition (DSM-5) criteria. The diagnoses were further confirmed by the principal investigator, and by the application of the direct structured proband assessment Autism Diagnostic Observation Schedule (ADOS).^38,39^ by two certified psychologists. All participants of the autism group had positive results in ADOS and met the DSM-5 criteria for an ASD diagnosis. Please note that the ADOS subscale scores for Repetitive Behaviours may have been underestimated due to inherent constraints related to the evaluation setting and duration. Specifically, given that the instrument captures only the behaviours expressed during the observation period, this methodological limitation may account for the obtained scores of 1 ± 0.77 (*mean* ± *standard deviation*). Participants in the neurotypical group were recruited through local social media campaigns.

Individuals were excluded from both groups if they presented with neurological or genetic conditions, intellectual disabilities, or psychiatric disorders that impact brain development, such as ADHD or epilepsy. Additional exclusion factors comprised substance or behavioural addictions, a history of significant head trauma, or any contraindications and abnormalities found during structural MRI screenings. Conversely, individuals in the autism group could remain eligible despite past diagnoses or subclinical symptoms associated with or secondary to ASD (for detailed information, see Supplementary Table 1). Ten participants from this group were doing psychopharmacological medication.

The Portuguese version 5.0.0 of the Mini-International Neuropsychiatric Interview (MINI) for DSM-IV,^40^ and alongside the Wechsler Adult Intelligence Scale—third edition (WAIS-III)^41,42^ were applied to all participants. Regarding cognitive performance, the neurotypical group showed a mean Full-scale IQ of 126.33 (±11.56), while the autism group presented a mean of 102.67 (±14.32).

This research adhered to the Declaration of Helsinki and received formal clearance from the local Research Ethics Committee (CHUC-089-20). Following a thorough briefing on the study's protocols, every participant provided written informed consent. To minimize participant burden, assessments were coordinated with existing hospital appointments.

### Stop-signal task

**Figure 1:**
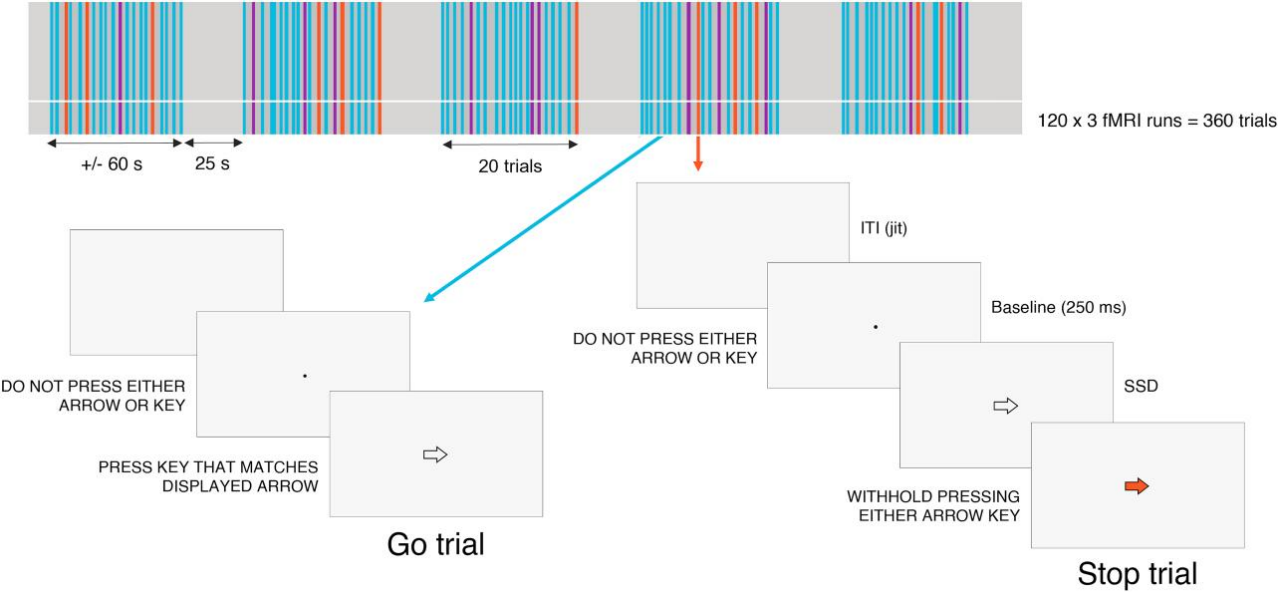
Schematic of the SST. The task included 3 runs of 120 trials (a total of 360 trials). Within each run, the trials were presented in blocks of 20 repetitions (60 s) interleaved with a baseline/response preparation period of 25 s. Go trials (75%) began with a fixation dot (250 milliseconds) followed by a go-signal, which was a white arrow either pointing to the right or left side on the screen, instructing participants to press the left or right button of the response box. Stop trials (25%) began with a fixation dot (250 ms) followed by the white arrow, which turned red (stop signal) after a variable period of time (stop signal delay), instructing the subject to withhold the response. Approximately half of the stop trials could not be stopped, as a result of the staircase implementation. The inter-trial interval (time between the end of the previous trial and the start of the current one) was jittered between 750 and 2750 ms. ITI, inter-trial interval; jit, jittered; SSD, stop-signal delay.

The SST employed in this fMRI study (illustrated in Fig. 1) was developed using Psychophysics Toolbox 3 in MatLab R2019b (The MathWorks, Inc., USA), adhering to standard protocols.^26,27,43^ The methodology for this task has been described in our previous papers^33,34^ and is here detailed as in Supplementary material.

In accordance with the independent race model,^27^ the *stop-signal reaction time* was estimated using Verbruggen’s integration technique.^26^ Other performance indicators,^25,27^ included in our analysis were the *go reaction time* (mean reaction time on go trials), *stop signal delay*, number of *omission go*’s (number of non-responses on go trials in relation to the number of total trials), probability of *successful stopping* (probability of stopping on stop trials). These metrics collectively characterize the behavioural profile of each group within the SST framework. The *stop-signal reaction time* serves as a quantitative measure of the time required to cancel an initiated prepotent response.^25^ Shorter *stop-signal reaction times* reflect higher effectiveness of the stopping mechanism.^27^ Achieving this requires participants to optimize their speed-accuracy trade-off, weighing rapid execution against the proactive slowing necessary for accuracy. Such strategic adjustments are manifested through longer *stop-signal delays*.^44,45^

To account for hypothesized strategic differences in inhibitory control within the autism group, we applied a liberalized *omission go* threshold of 15% for ‘go’ trials, which is more lenient than the standard 5% exclusion criterion recommended for neurotypical individuals.

### MRI procedure

The complete MRI session last 60-minute, while the SST was applied during the final 30 min of the MRI protocol. The stimuli were visualized on a high-definition LCD monitor (48.5 × 87.8 cm, 1920 × 1080 pixel resolution, 60 Hz refresh rate) through a mirror mounted above participant’s eyes. The distances from the participant’s eyes to the monitor superior and inferior limits were around 1750 mm and 1825 mm, respectively. The individuals could respond to the task via an MRI-compatible pad. All participants were right-handed and performed the task using their dominant hand. MRI-compatible lenses were provided to participants requiring visual correction to ensure normal or corrected-to-normal vision.

### fMRI acquisition and preprocessing

MRI data were acquired using a 3 Tesla Magnetom Prisma Fit scanner (Siemens, Erlangen, Germany), equipped with a 64-channel head coil. The protocol began with one structural MPRAGE sequence, with a repetition time (TR) = 2530 ms, echo time (TE) = 3.5 ms, resolution 1 mm^3^, flip angle = 7°, 192 slices and field of view (FOV) = 256 × 256 mm. The functional EPI sequences were acquired with a multi-band acceleration factor of 6, a voxel size of 2 × 2 × 2 mm^3^, 72 interleaved slices with 0% spacing, A-P phase encoding, slices parallel to the AC-PC line, TR of 1000 ms, TE of 37 ms, flip angle of 68° and FOV of 200 × 200.

Imaging data were pre-processed using BrainVoyager versions 22.0 and 22.4 (Brain Innovation, Maastricht, The Netherlands). Consistency in algorithms across versions ensured that procedural reliability remained uncompromised. A standard pipeline was applied, comprising slice-scan time correction, motion correction in the three axes, and filtering in the time domain using a high-pass filter (2 cycles per run). Geometric distortions were corrected using the BrainVoyager plugin, COPE.^46^ The resulting functional volumes were co-registered with the individual structural images and normalized to the MNI space. After spatial normalization, smoothing was done using a Gaussian kernel of 4 mm FWHM. Runs with head motion exceeding 6 mm along any axis were discarded, resulting in the exclusion of 17 runs (9 neurotypical; 8 autism). The average framewise displacement across the 3 runs was 0.131, 0.123 and 0.126 mm in the neurotypical group, and 0.198, 0.21 and 0.241 in the autism group. Motion parameters and physiological noise (respiratory and cardiac signals, generated via the PhysIO toolbox for SPM in Matlab^47,48^) were incorporated as nuisance regressors in the GLM.

### Self-report questionnaires

To assess non-clinical autism-spectrum traits and the global severity of RBs in the previous month and childhood period (0–12 years), participants and their close relatives (*e.g.* a family member) completed the Portuguese versions of the *Autism Spectrum Quotient*^49^ and *Repetitive Behaviour Scale-Revised* (*RBS-R*).^50^ These instruments are primarily screening tools; however, higher scoring levels may signal the requirement for a more comprehensive diagnostic investigation. Detailed descriptions of the questionnaires are provided in Supplementary material. As predicted, participants with autism exhibited higher scores across all domains. Due to missing data, it was not possible to calculate (a) the total *Autism Spectrum Quotient* scores for 7 out of 18 subjects with autism, (b) the *RBS-R* adulthood scores for 1 out of 21 neurotypical subjects, and 2 out of 18 subjects with autism, and (c) the *RBS-R* childhood scores for 1 out of 21 neurotypical participants, and 4 out of 18 participants with autism.

### Data analysis

#### Brain activity

Functional neuroimaging data were analyzed using Brain Voyager software, versions 22.0 and 22.4 (Brain Innovation, Maastricht, the Netherlands). At the first level, a GLM was implemented for each participant. Predictors were generated by convolving the time courses of each experimental condition with a canonical two-gamma haemodynamic response function. All regressors were modelled as events with a duration of 1 TR (1 s), time-locked to the onset of the stimulus presentation (the appearance of the left- or right-pointing arrows).

In accordance with recent consensus work^26^ and our hypotheses, the GLM included five distinct trial-based predictors: *Correct Go* (successful responses to directional arrows), *Successful Stop* (effective response inhibition during stop trials), *Failed Stop* (unsuccessful inhibition), *Post-error processing* (the interval separating a failed stop trial from the following trial), and *Post-hit processing* (the interval separating a correct stop/correct go trial from the following trial, excluding instances defined as *Post-error-processing*). Please note that in addition to standard SST measures, this study incorporated *Post-hit* and *Post-error processing* to capture brain activity related to predicted learning and outcome valuation following a response.^36,45^  *Baseline/Response Preparation* refer to periods preceding stimulus-presentation.

Group-level RFX analyses were performed to map error-related whole-brain activation (*Failed Stop*  *>*  *Baseline/Response Preparation*; RFX, *t*(39) = 4.22, *P*-FDR < 0.001). This specific localizer enabled the detection of regional activity changes related to inhibition errors, highlighting the role of basal ganglia and dopaminergic midbrain regions as predicted by integrated models of inhibition. Activation maps derived from contrasting *Failed Stop* against *Baseline/Response Preparation* periods are displayed in Supplementary Fig. 1, while the corresponding cluster coordinates and peak intensities are reported in Supplementary Table 2.

**Figure 2:**
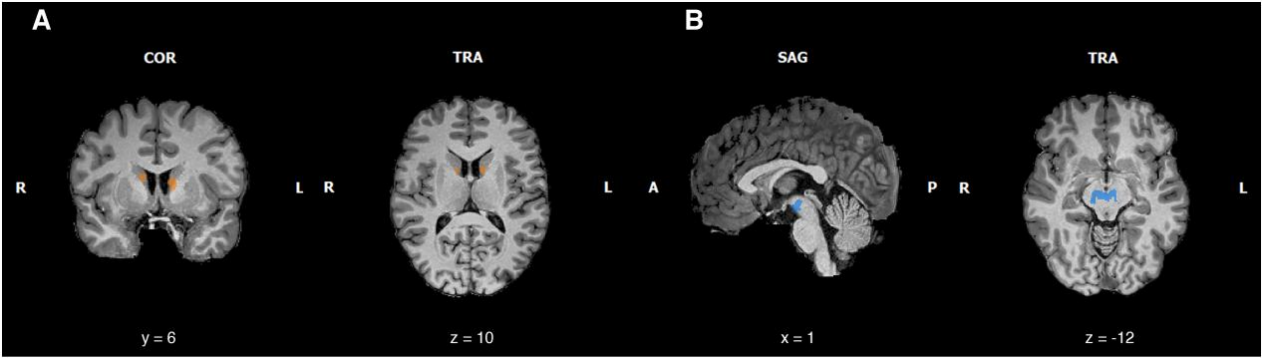
Regions of interest based on the actor-critic model used for the main analysis. ROIs to investigate error-related inhibitory processes in autism were defined in the striatum (**A**) according to our whole-brain activations (*Failed Stop* > *Baseline/Response Preparation*; RFX, *t*(38) = 6.14, *P*-Bonferroni = 0.1); and in the midbrain (**B**) using the intersection between our whole-brain activations (*Failed Stop*  *>*  *Baseline*; RFX *t*(38) = 3.80, *P-FDR* < 0.005) and anatomical boundaries.^51,52^ Multiple comparisons correction was set to a minimum threshold that allowed us to isolate our ROIs from the larger clusters and taking into account the localization nature of this independent analysis, such that smaller structures (those in the midbrain) required a more liberal threshold. COR, coronal; FDR, false discovery rate; RFX, random effects; SAG, sagittal; TRA, transversal.

We delineated ROIs in the midbrain and basal ganglia by combining functional and anatomical constraints. Specifically, we used the intersection of the significant RFX functional clusters and standardized anatomical masks.^51,52^ Regions of interest (ROI) in the midbrain and basal ganglia were delineated based on the resulting clusters and our previous work substantiating the ‘actor-critic’ framework in healthy humans^33^ and individuals with obsessive-compulsive disorder.^34^ Both anatomical and functional criteria were considered by mapping the intersection of our significant RFX activation map and established anatomical boundaries of these regions.^51,52^ The subthalamic nucleus, globus pallidus, and nucleus accumbens, were discarded from further consideration due to the absence of significant cluster signalling. ROIs in the basal ganglia were defined directly from our whole-brain maps (*Failed Stop*  *>*  *Baseline/Response Preparation*; RFX, *t*(38) = 6.14, *P-Bonferroni* = 0.1), resulting in a cluster in the dorsal striatum—*i.e.* the ‘actor’—including the head of the caudate and putamen (Fig. 2A). To isolate dopaminergic midbrain regions, we intersected our whole-brain maps (*Failed Stop* > *Baseline/Response Preparation*; RFX *t*(38) = 3.80, *P*-FDR < 0.005) with two probabilistic atlases^51,52^ from the Adcock Lab (Duke University). This procedure yielded one ROI encompassing the lateral VTA and SN (Fig. 2B), corresponding to the ‘critic’. Thresholding was strategically adjusted—applying more liberal criteria to the smaller midbrain structures—to successfully dissociate these functional units from surrounding clusters while preserving the independence of the localizer approach.

Based on our previous investigation on the ‘actor-critic’ framework,^33,34^ and consensus in the SST field,^36^ beta-values were derived for two contrasts of interest representing failed inhibition (*Failed Stop* > *Correct Go*) (Korucuoglu *et al*.^53^), and subsequent error processing (*Post-error processing* > *Post-hit processing*). This allowed us to examine how the dopaminergic-striatal system encodes outcome valuation and strategic adjustment during and in the aftermath of inhibition errors.

#### Functional connectivity

Functional connectivity analyses were conducted using the CONN toolbox (version 22a) in MATLAB 2020b (MathWorks®).^54,55^

First, anatomical and functional data were preprocessed following the standard procedure^54^ and applying spatial smoothing of 4 mm to match activity mapping analysis. Then, a denoising step was applied to mitigate potential confounding effects (10 components from white matter and CSF, 12 components from motion correction and their 1st order derivatives, 162 components from identified outlying scans, and 4 components for task effect and their 1st order derivatives). Linear detrending was applied and data were high-pass filtered at 0.008 Hz minimizing low-frequency drift effects.

To characterize task-dependent variations in functional connectivity variation patterns, we estimated seed-based and ROI-to-ROI metrics using the generalized psychophysiological interactions (gPPI) framework.^56^ The fast nature of our design limited the precise temporal dissociation of inhibitory failure from subsequent error processing neural correlates. So, functional connectivity estimates were based on response preparation. A separate multiple regression model was fitted to each target voxel or ROI BOLD time series to compute gPPI effects. Each model included three independent regressors: the task effect convolved with a canonical haemodynamic response function, the seed ROI time series, and their interaction, defined as the product of the first two regressors. gPPI results were expressed as the regression coefficients associated with this interaction term. GLM-based group analyses were carried out separately for each voxel or ROI, using first-level connectivity estimates as dependent variables, group as the independent variable, and IQ as a second-level covariate. Group-level seed-based connectivity analyses relied on Gaussian Random Field theory-based parametric statistics^57^ applying a voxel-wise cluster-forming threshold of *P* < 0.001 and a familywise FDR-corrected cluster-level threshold of *P* < 0.05. For visualization purposes, individual subject values were adjusted for the covariate (IQ) using the second-level GLM. Adjusted values were computed as the model-estimated group mean plus the subject-specific residual, thereby preserving inter-individual variability while ensuring consistency with the estimated marginal means. Group-level ROI-to-ROI connectivity was examined using multivariate parametric functional network analyses,^58^ with significance established via an *FDR*-corrected familywise cluster-level threshold (*P* < 0.05) and an uncorrected *post hoc* connection-level threshold (*P* < 0.05).

#### Statistical analysis

Statistical procedures were carried out using the Statistical Package for Social Sciences, version 29 (SPSS ®, Chicago, IL, USA). Between-group differences in SST performance were assessed across five key parameters: *go reaction time*, *stop signal delay*, *omission go*, *successful stopping*, *stop signal reaction time* (SSRT). To investigate whether group-specific activation in the striatum and midbrain was modulated by the *inhibition* phase, a Mixed Repeated Measures ANCOVA was conducted. The model included ROI (striatum and midbrain) and *inhibition* phase (failed inhibition and error processing) as within-subjects factors, group (autism and neurotypical) as between-subjects factor, and IQ as co-variate.

To identify the variables contributing significantly to the model, *post hoc* independent *t*-tests were conducted. We subsequently examined the correlations between neural measures (activity and connectivity) and (a) task performance, within each group, and (b) RB symptoms, in the autism group; all controlled for the effect of IQ level. All the statistics were FDR-corrected at a *P* level of 0.05. To further elucidate the influence of midbrain activation on RB severity in autism, we performed mediation analysis using PROCESS macro (Model 4) for SPSS.^59^ Only variables that demonstrated significant effects in the preceding analyses were inserted into the mediation model.

## Results

### Stop-signal task behavioural performance

Group comparison revealed similar task performance between participants with autism and neurotypical subjects. The two groups adhered to the task rules, as indicated by the low rate of *omission go* trials [autism: 2.51 ± 3.34% versus neurotypical: 1.22 ± 2.7%, *Z*(1, 36) = 1.037, *P* = 0.315]. In around half of the stop trials, both subjects with autism [52.99 ± 4.84%, *Z*(1, 36) = 0.765] and neurotypicals (51.95 ± 3.39%, *P* = 0.388) were able to successfully withhold their response (*Successful Stop*), while in the other half, they failed to stop (*Failed Stop*), which reflects the effective operation of the staircase procedure. SSRT scores in both groups [autism: 247.82 ± 39.49 ms versus neurotypical: 262.5 ± 48.04 ms, *Z* (1, 36) = 0.680, *P* = 0.415] were within the expected range for healthy adults,^60^ being consistent with previous literature reports.^61^

These findings indicate that adults with autism exhibit inhibitory performance levels comparable to neurotypical counterparts. Detailed information regarding the participants’ performance on the SST is presented in Supplementary Table 3 and Supplementary Fig. 2.

### Striatum and dopaminergic midbrain recruitment during the SST

ROI analysis was focused on error-related neural activity responses in the basal ganglia and dopaminergic midbrain regions, based on the hypotheses that (a) individuals with autism have altered mechanisms of processing errors, particularly, those occurring during response inhibition failures, and (b) striatal and midbrain structures are implicated in this autism-specific information processing modes. Whole-brain GLM analysis including the entire sample (neurotypical + autism groups), for the contrast *Failed Stop* > *Baseline/Response Preparation*, confirmed that error commission in our SST recruited regions within the hypothesized ROIs, which were further validated with anatomical criteria.^51,52,62^ For detailed information regarding the strategy to define our relevant ROIs see the Methods section, Supplementary Fig. 1, and Supplementary Table 2. Then, we extracted *beta*-values from the dorsal striatum (caudate + putamen; the ‘actor’; Fig. 2A) and midbrain (VTA + SN; the ‘critic’; Fig. 2B) for our contrasts of interest: failed inhibition (*Failed Stop* > *Correct Go*) and error processing (*Post-error processing* > *Post-hit processing*). Region size and peak voxels in standardized MNI space for the identified ROIs were as follows: striatum: 1035 voxels, *x* = −10, *y* = 8, *z* = 8; midbrain: 1362 voxels, *x* = −4, *y* = −18, *z* = −15.

### Effect of inhibition phase on autism-related activation changes in the striatum and midbrain

**Figure 3:**
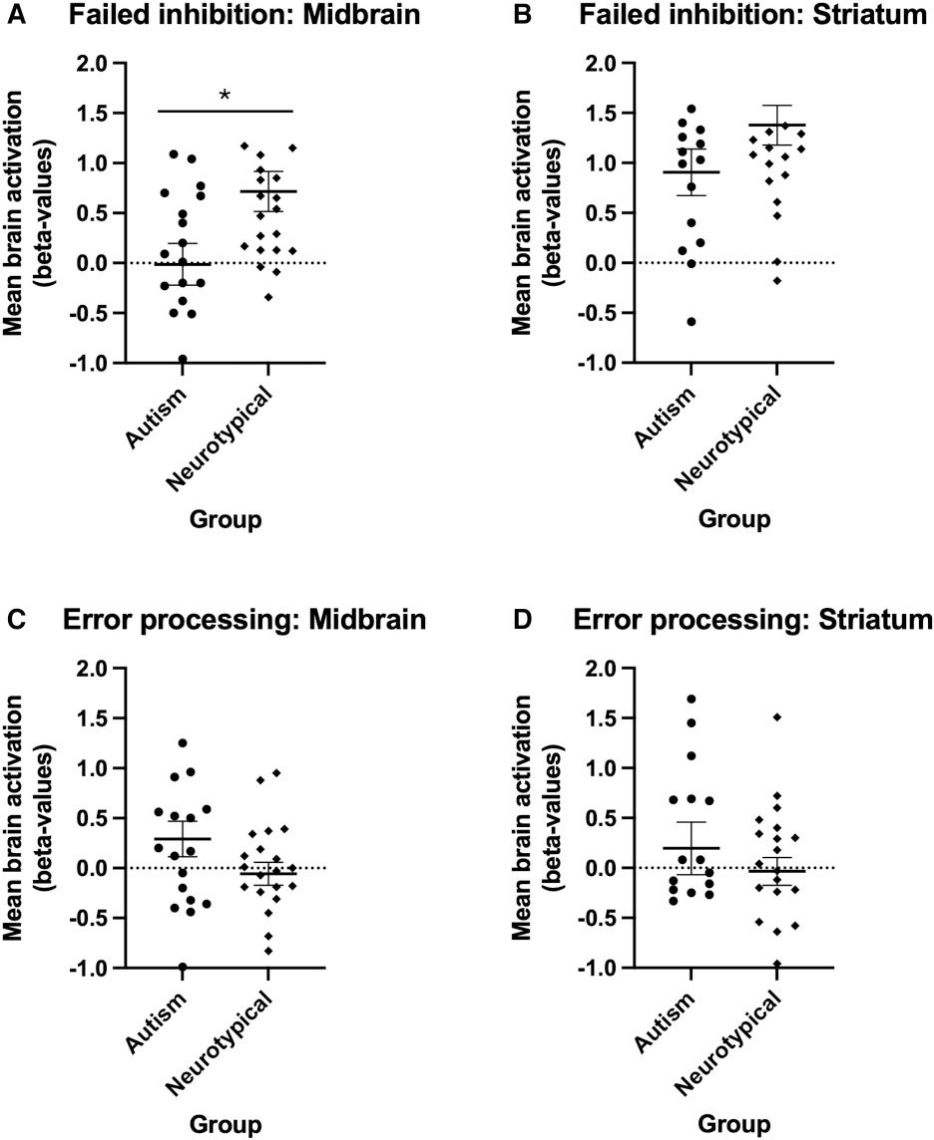
Mean brain activations (beta-values) in the midbrain and striatum, related to failed inhibition (**A** and **B**) and error processing (**C** and **D**) in individuals with autism (*n* = 18) and neurotypical subjects (*n* = 21). Error bars represent the standard error of the mean. Between-group comparison using *IQ* and risperidone equivalents as covariates showed midbrain hypoactivation related to failed inhibition in the autism group (*F*(1, 35) = 9.165, *P* = 0.005; see * symbol in 3A), which therefore was the main effect contributing to significant group × inhibition phase interaction in the first-order model (*Z*(1, 36) = 6.981, *P* = 0.012). IQ: intelligence quotient.

To investigate activation differences between the autism and neurotypical groups in the ‘actor-critic’ regions and their dependency on specific inhibition subdomains, we applied the Mixed Repeated Measures ANCOVA. The first-order model, with *inhibition* phase (failed inhibition and error processing) and ROI (midbrain and striatum) as within-subjects factors, group (autism versus neurotypical) as between-subjects factor, and IQ and antipsychotic use (risperidone equivalents) as co-variates showed a significant interaction between group and *inhibition* phase (*F*(1, 35) = 9.165, *P* = 0.005; Supplementary Table 4.1). Results showed no significant effect of medication use (*F* = 0.712, *P* = 0.405). *Post-hoc* between-group comparison (Fig. 3; Supplementary Table 4.2) revealed that the significant effect in our model mainly resulted from midbrain activation differences related to failed inhibition (*Z*(1, 36) = 5.218, *P* = 0.028), such that there was hypoactivation (and deactivation) in the autism group (*M* = −0.036, SD = 0.860), compared with the neurotypical one (*M* = 0.715, SD = 0.918).

These results point to midbrain regions as functionally relevant during neural *inhibition* processes in autism, with a major role during error commission.

### Autism-related functional connectivity changes in the ‘actor-critic’ network

**Figure 4:**
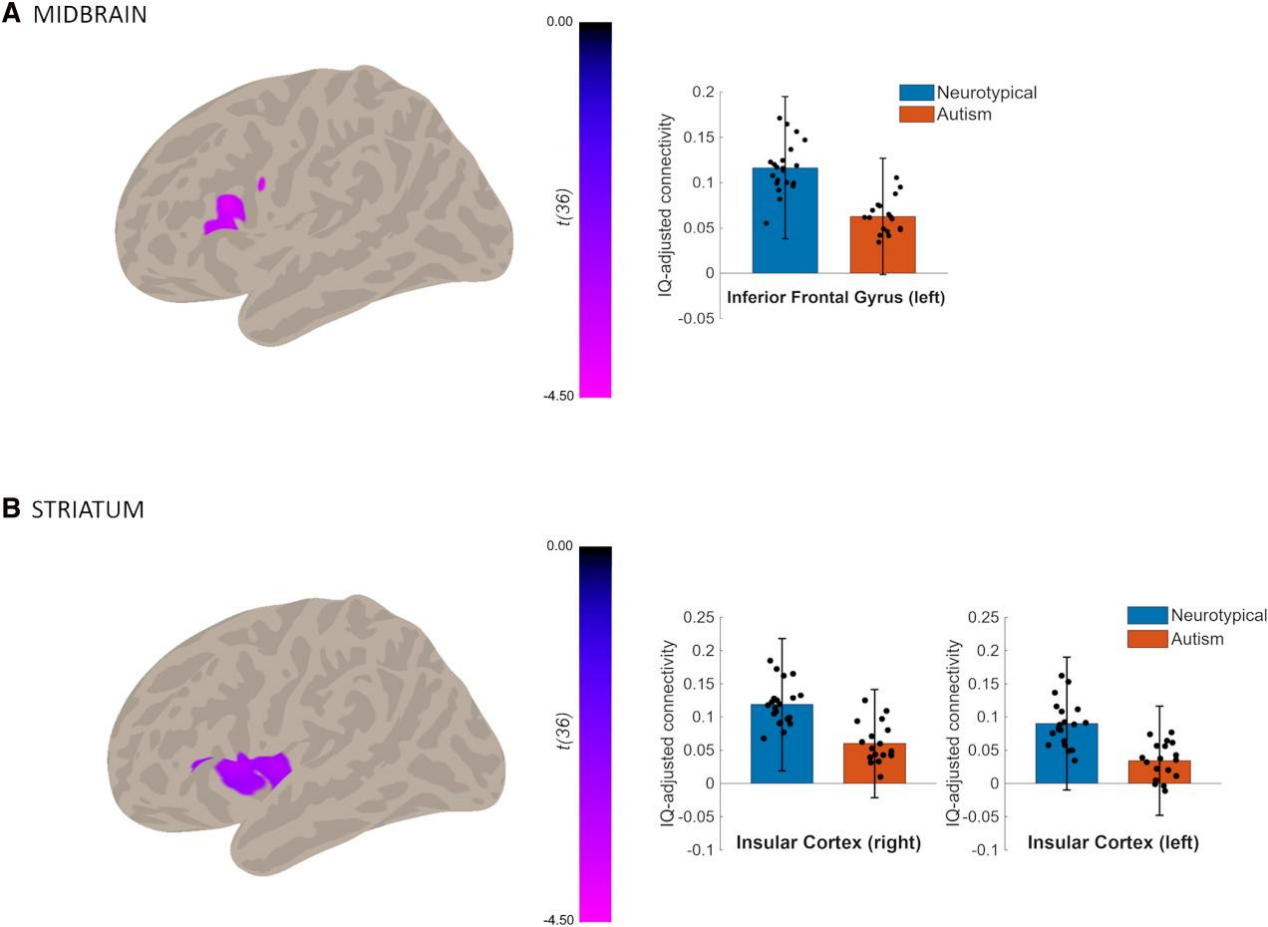
gPPI whole-brain functional connectivity analysis in autism (*n* = 18) and neurotypical (*n* = 21) groups when considering as seed region the midbrain (**A**) and striatum (**B**). The colour scale represents the strength of the *t*-statistic, indicating the difference in connectivity between autism and neurotypical subjects from response inhibition to response preparation, which was found to be decreased in autism. The differences in functional connectivity between each seed region and the rest of the brain are displayed at a voxel-level threshold of *P* = 0.001 and a cluster-size threshold of *P*-FDR = 0.05. Bars represent estimated marginal means of seed-to-voxel connectivity for each group, adjusted for IQ. Error bars indicate ± 1 standard error of the mean. Dots represent individual subjects’ IQ-adjusted connectivity values, reflecting residual variability around the group mean. FDR, false discovery rate.

To investigate if functional connectivity between ‘actor-critic’ nodes and all other brain regions varied between groups, from response inhibition to response preparation (*i.e.* the period when proactive control mechanism for response suppression are expected to engage), we ran a seed-based gPPI analysis. As shown in Fig. 4, individuals with autism exhibited reduced connectivity between the midbrain and a cluster centred in the left inferior frontal gyrus (MNI coordinates: *x* = −56, *y* = 14, *z* = 22; *t* = −4.94, *P*-FDR = 0.00003); and between the striatum and two clusters centred in the insular cortex (MNI coordinates: *x* = −37, *y* = −12, *z* = 4 and *x* = 36, *y* = 22, *z* = 4), in the left and right hemispheres (*t* = −4.00, *P*-FDR = 0.0003, and *t* = −4.43, *P*-FDR = 0.00009, respectively). ROI-to-ROI analysis using the striatum and midbrain as seeds did not reveal significant functional connectivity differences between groups.

### Associations between repetitive behaviours and neural inhibition in the striatum and midbrain

The severity of RBs in the autism group was measured by the informant-based *RBS-R* regarding both the adulthood (previous month) and childhood (from 0 to 12 years of age) periods. We found significant correlations between symptom severity and decreased error processing activity in the midbrain (adulthood: *r* = −0.581, *P*-FDR = 0.046; childhood: *r* = −0.621, *P*-FDR = 0.046) and striatum (adulthood: *r* = −0.639, *P*-FDR = 0.04; childhood: *r* = −0.747, *P*-FDR = 0.024; all controlled for the effect of IQ level; see Supplementary Table 5 for detailed information).

**Figure 5:**
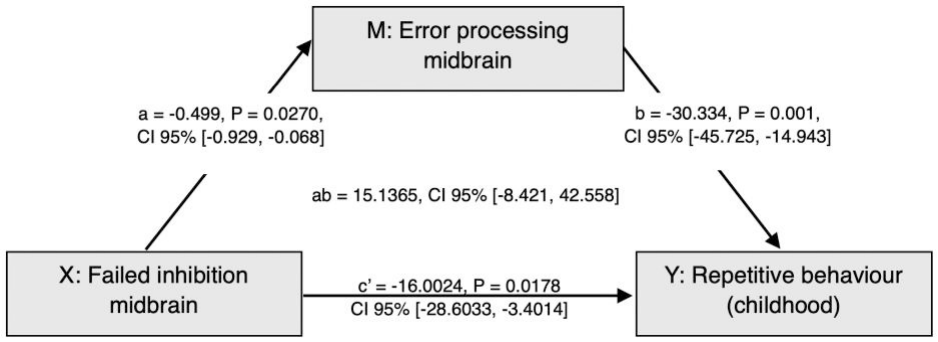
Effect of the relationship between failed inhibition and error processing activity in the midbrain on childhood repetitive symptom severity in individuals with autism (*n* = 14), using IQ as co-variate. Serial multiple mediation model (Model 4 of the PROCESS macro for SPSS) showing a significant indirect effect of failed inhibition-related midbrain activity on autism repetitive behaviours severity (childhood period) mediated by error processing activity in the same region. Numbers represent unstandardized coefficients. *X*: predictor variable; *M*: mediator; *Y*: outcome variable; *a*: effect of *X* on *Y*; *b*: effect of M on Y; *ab*: indirect effect of *X* on *Y*; *c*’ direct effect of *X* on *Y*; CI, confidence interval.

Considering our results in the autism group of (a) midbrain hypoactivation during failed inhibition, and (b) midbrain deactivation-activation across failed inhibition and error processing, instead of the activation-deactivation sequence exhibited by neurotypical subjects (Fig. 3), we sought to further investigate the role of the midbrain on RBs, based on the hypothesis of a late adaptation mechanism. Accordingly, we performed mediational analyses to test the effect of error processing activity in the midbrain on the relationship between failed inhibition activity in this region and RB symptoms. In fact, controlling for the effect of error processing activity (mediator variable), decreasing failed inhibition midbrain activity (predictor variable) predicted more severe childhood RB symptoms [outcome variable; *c*’ = −16.0024, *P* = 0.0178, CI 95% (−28.603, −3.401); Fig. 5], and thus supported our hypothesis. Regarding adult RBs, the direct, indirect, and total effects of midbrain activity on symptoms were all non-significant.

### Associations between learning and neural inhibition in the striatum and midbrain

**Figure 6:**
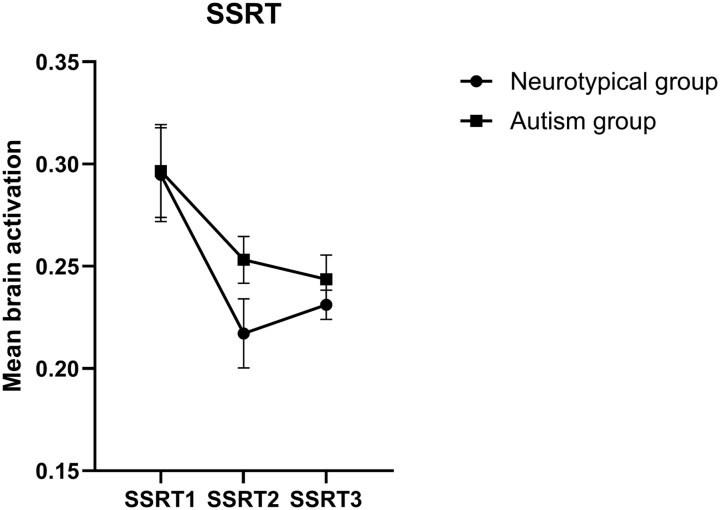
Evolution of SSRT scores during the task in autism and neurotypical groups. The figure shows a decrease in the SSRT scores throughout the task, suggesting performance improvement dependent on trial-and-error learning. Significant changes were observed from run 1 to run 3, both in neurotypical subjects (*M* = 0.064 ms, SD = 0.09 ms, *t*(14) = 2.591, *P* = 0.021, *n* = 21), and individuals with autism (*M* = 0.06 ms, SD = 0.06 ms, *t*(10) = 2.896, *P* = 0.016, *n* = 18). Error bars represent the standard error of the mean.

Regarding performance on the SST, we found decreasing SSRT scores over the three task runs in both groups, compatible with an improvement pattern within a trial-and-error learning framework (Fig. 6). ANCOVA analysis showed no effect of group on the stop-signal reaction time patterns. Then, to investigate if the ‘actor-critic’ network and regions were involved in inhibitory learning processes and strategies underlying the SST in each group, we investigated correlations between neural inhibition in the striatum and midbrain, and task performance using: (a) the SSRT, corresponding to the final task output, and (b) the *stop signal delay*, which reflects the strategical modulation of the stimulus-response rules to increase the likelihood of *successful stopping* at the cost of response time slowing (‘speed-accuracy trade-off’). Considering our previous study in healthy subjects,^33^ we hypothesized to find significant relationships between (a) brain connectivity and the SSRT, and (b) brain activity and the *stop signal delay*.

In the autism group, we identified a correlation between shorter mean SSRT scores and higher connectivity strength between the striatum and midbrain (*r* = −0.537, *P*-FDR = 0.05), which was not observed in neurotypical subjects. Longer *stop signal delay* scores presented significant correlations with striatal activity related to failed inhibition (*r* = 0.519, *P-*FDR = 0.038) and error processing (*r* = 0.524, *P*-FDR = 0.038) in neurotypical subjects; and error processing in subjects with autism (*r* = 0.619, *P*-FDR = 0.032). All the analyses were controlled for the effect of IQ level.

These results support the role of the ‘actor-critic’ network in inhibitory performance during the SST and suggest that strategical response slowing following negative feedback in autism relies on late striatal engagement.

## Discussion

We investigated the hypothesis that neural and behavioural inhibition in autism is based on condition-specific markers in the midbrain-striatum network. Our previous studies revealed distinct neural profiles in these ‘actor-critic’ brain regions in healthy adults^33^ and patients with obsessive-compulsive disorder.^34^ In individuals with autism, we identified error-related midbrain hypoactivation and mesocortical hypoconnectivity suggesting reductions in error valuation mechanisms and proactive control. This striking neuroimaging profile, which we observed under intact performance conditions, predicted the severity of RBs and was associated with delayed styles for error-learning.

Both groups (autism and neurotypical) were performance-matched and exhibited an improvement pattern in their inhibitory response times through the SST, supporting the successful involvement of learning mechanisms driven by repeated trial-and-error experiences. However, neurotypical subjects learned faster and engaged the striatum more promptly when response correction was required. While the notion that autistic people have difficulties in early learning^63^ and use different neural mechanisms to complete inhibitory tasks is expectable,^5,8^ the exact underpinnings of this relationship remained largely unexplored.

The most remarkable findings regarding these neural correlates were in dopaminergic brain regions. Participants with autism exhibited hypoactivation in the midbrain related to failed inhibition instances, which was not present during error processing. Neurotypically, midbrain activation during error events is thought to encode the estimation of an error signal that guides the dorsal striatum in action selection.^32,64-66^ Conversely, our results indicate that, in neurodivergent subjects, the prediction error signal driving reinforcement learning and motivation in the midbrain leads to suboptimal processing of the stop signal. Consequently, we highlight immediate error valuation (corresponding to the aftermath of stopping failures), as mediated by dopaminergic midbrain regions, as a pivotal process for understanding neurodiverse processing styles. Activity in the dorsal striatum was equivalent between the two groups across both inhibition phases, further emphasizing that midbrain dysfunction may constitute a primary source of impairment in autism during error-learning.

The ability to recognize errors and adjust behaviour accordingly also depends on the midbrain and striatum exchanging information with cortical regions.^33,67^ Participants with autism in the present study displayed reduced functional connectivity between the midbrain and regions involved in executive and cognitive control (left inferior frontal gyrus), as well as between the dorsal striatum and salience processing regions (anterior insula), both during response preparation phases. The prefrontal and insular cortices, due to their functions in top-down cognitive control, and high-level neural network coordination, attentional and sensory-motor processing have been extensively implicated in social and non-social dimensions of autism.^3^ However, there remains no consensus on the precise role of these regions.^68-70^ Our study demonstrates midbrain and striatal hypoconnectivity in autism in periods preceding action instrumentalization, where proactive inhibition is required.^14^ Schmitt *et al.*^61^ in a large cohort of subjects with autism, found a selective deﬁcit in proactive control strategies, which scaled with the severity of RBs. Mirabella *et al.*^23^ proposed that proactive inhibition difficulties in this condition could clinically manifest in an inability to learn using contextual cues to inhibit inappropriate RBs. So, considering the midbrain and striatum roles in exploiting external and internal cues to choose the most appropriate actions, it is plausible that hypoconnectivity in these regions contributes to proactive control alterations displayed by individuals with autism. Functional connectivity between the midbrain and striatum remained intact in the neurodivergent group, coinciding with the prevailing view that autism primarily affects long-range connectivity.^6,71-74^ Connectivity strength within this midbrain-striatum network was associated with enhanced inhibitory performance, which aligns with findings from Di Martino *et al.*^69^ These authors identified a similar link in which striatal hyperconnectivity with the pons was associated with reduced RBs in children with autism. Accordingly, we suggest that reliance on preserved or enhanced regional connectivity within the ‘actor-critic’ network may represent an adaptation response in autism offsetting inefficient long-range communication.

These data highlight an autism phenotype marked by error insensitivity, reduced long-range dopaminergic signalling during proactive inhibition, and delayed neurocognitive styles for response correction. The expected consequence is an overreliance on reactive (habit-driven) inhibition modes, leading to last-minute error detection and control implementation, as previously demonstrated in autism regarding probabilistic decision-making,^1,2,22^ and supported by numerous findings of increased latencies and reduced amplitude of the error-related negativity.^19,75,76^ So, reduced midbrain activation during error events emerges as a potential neuroimaging parallel of these behavioural and electrophysiological markers of autism. Reactive inhibition, although effortless and fast, commonly leads to automatic, repetitive, and inflexible behaviour.^14^ Here, we observed a relationship between observer-reported RBs in individuals with autism and decreased midbrain and striatal activity during inhibition failures. These findings provide evidence for a neurobehavioural link between midbrain hypoactivation (and the presumably resulting motivational impairment) and a tendency to perseverate on out-of-context and outcome-insensitive RBs. Subsequent activity increases in the midbrain-striatum axis are compatible with a late adaptation mechanism for response adjustment and symptom attenuation. So, the same mechanisms applied by subjects with autism for completing the SST, based on reduced and delayed midbrain and striatal engagement, were also significant for symptom manifestations.

Our study remarkably positions dopaminergic midbrain structures as pivotal in the neurobiology of autism. A view centred on dopaminergic subcortical regions provides new insights that depart from traditional conceptualizations of autism as a cortico-cortical disconnection syndrome and complement recent large-scale findings of subcortical-cortical hypoconnectivity in this condition.^68,77,78^ Notably, brainstem-focused perspectives^79^ have garnered significant evidence from earlier neuropathological ,^80^ morphological^81-83^ and neurophysiological^84^ investigations. Drawing on the developmental ontogeny of the brain, this alternative hypothesis posits that primary brainstem injury propagates toward the cerebral cortex, resulting in large-scale disruption of neural circuitries underlying autism phenotypes.^73,85^ Similarly, ‘social motivation’^86^ and ‘dopaminergic’^87^ theories posit that social difficulties and RBs associated with autism arise from reduced dopaminergic functioning in the mesocorticolimbic and nigrostriatal pathways. However, the general concept of a hypofunctional reward system does not provide a detailed or integrated mechanistic model. A significant conundrum concerns the established physiopathology of RBs in other circumstances, such as amphetamine-induced RBs, tics, and dyskinesias, as these conditions are frequently linked to elevated, rather than decreased, striatal dopamine levels.^11,15^ Our results indicate that, contrary to impulsive-compulsive disorders, which encompass disinhibition towards goal-directed and/or habitual behaviours,^11,15^ RBs in autism result from error valuation difficulties and reliance on more habit-like ways of responding. Reduced error valuation disrupts the formation of stimulus-response-outcome associations^88^ necessary for the development of a reinforcement history and flexible strategy adjustment. Therefore, in autism, responses to inhibitory cues can be interpreted as more similar to fast, redundant, and inflexible trial-by-trial reactions that do not require prior learning. Impairments in motivation and proactive control are consistent with the notion that neurodivergent individuals are slower learners, but once automatic performance modes are achieved, they may experience intense feelings of reward during task completion.

Additionally, the association between midbrain hypoactivation and observer-reported RBs suggests a crucial relationship between error insensitivity and symptom severity. Therefore, autistic RBs should stem from reduced motivational arousal, contrasting with the hyperarousal seen in other conditions characterized by RBs, such as obsessive-compulsive disorder.^34^ In animal studies, partial dopamine depletion results in reduced energy for learned tasks.^89^ Conversely, restoring dopamine synthesis re-establishes locomotion and food-seeking in dopamine-deficient mice.^90-92^ Our research, in humans, similarly indicates that hypofunction in dopaminergic brain regions interferes with the ability to learn that context-inappropriate behaviours should be inhibited. This is consistent with prior work from our group suggesting the relevance of dopaminergic gene dosage effects in autism.^93^

Autism models based on dopaminergic dysregulation should also explain important atypicalities in treatment outcomes of dysfunctional RBs and other comorbid symptoms (*e.g.* anxiety, irritability), namely (a) lower response rates to antidepressants, such as selective serotonin reuptake inhibitors, which down-regulate arousal mechanisms, (b) response to psychostimulant (dopaminergic) medication in some cases, and (c) higher vulnerability to extrapyramidal symptoms with first- and second-generation antipsychotics that block D2 receptors. Accordingly, we suggest that future therapies, like third-generation antipsychotics (*e.g.* aripiprazole, brexpiprazole, and cariprazine), should focus on the balance between dopamine agonism and antagonism. Neuromodulation therapies that increase dopamine release may also represent a promising approach to improving certain executive difficulties experienced by individuals with autism.^94^

A limitation of our work was the focus on adult male participants, implying that reproducibility requires replication in larger and more diverse samples, including children and individuals of the female sex. Additionally, the correlation between midbrain activity during failed inhibition and symptom severity warrants further consideration, as it was significant regarding childhood assessments but not adulthood. A plausible explanation is that childhood RBs, being simpler and more akin to biological models of impulsivity, involve fewer intervening factors compared with more complex adult behaviours.^15^

In conclusion, our investigation confirms the hypothesis that the actor-critic (striatum-midbrain) framework can explain inhibitory control phenotypes in autism. The main finding is the large hypoactivation and hypoconnectivity of value-based dopaminergic midbrain regions during immediate error valuation and proactive response preparation. Functional activity and intrinsic connectivity in the midbrain-striatum were respectively related to RB regulation and learning (as indexed by the final inhibitory output of the SST) in the autism group. This evidence suggests that individuals with autism exhibit midbrain-dependent hypofunctional error valuation mechanisms, limiting their ability to develop proactive strategies for trial-and-error learning and having to rely on trial-by-trial reactive response styles. By highlighting the key role of dopaminergic midbrain regions in the learning patterns of neurodivergent individuals, we challenge the prevailing view of autism as a dominant cortical dysfunction syndrome. In this line, midbrain-mediated neural pathways emerge as promising targets for neurobiologically-driven therapies aimed at improving RBs.

## Supplementary Material

fcag265_Supplementary_Data

## Data Availability

The data supporting the findings of this study are available from the corresponding author on legitimate request, though they are not publicly accessible due to legal and ethical constraints regarding participant privacy.

## Declaration of AI and AI-assisted technologies in the writing process

Generative artificial intelligence (Large Language Models) was utilized during the preparation of this manuscript to improve the linguistic quality, grammatical accuracy, and stylistic clarity of the text. The AI was not used to generate original scientific hypotheses, conduct data analysis, or interpret the neuroimaging and clinical results. All technical content, including the specification of the fMRI models, the ‘actor-critic’ theoretical framework, and the interpretation of the autism-specific findings, was authored and critically appraised by the research team. The authors take full responsibility for the integrity of the final manuscript and ensure that all AI-assisted suggestions were meticulously verified for scientific accuracy.
