# Supplementary material for: Midbrain hypoactivation and mesocortical hypoconnectivity in inhibitory control learning in autism
Source: Brain Commun. 2026 Jul 12;8(4):fcag265. doi: 10.1093/braincomms/fcag265 (PMC13403574; doi:10.1093/braincomms/fcag265)
Supplement: fcag265_Supplementary_Data [file fcag265_supplementary_data.pdf]

Midbrain hypoactivation and mesocortical hypoconnectivity in inhibitory control learning in autism

Ana Araújo,[1,2,3,4,5] Isabel C. Duarte,[1,2] Teresa Sousa, [1,2] Sofia Meneses,[6] Ana T. Pereira,[1,2,3] Trevor Robbins,[7] António Macedo,[1,2,3,4,5] and Miguel Castelo-Branco[1,2,4]

Supplementary material

Supplementary Table 1 Participants' demographic and clinical characteristics

	**Neurotypical** (*n* = 21) *Mean* (*SD*)	**Autism** (*n* = 18) *Mean* (*SD*)
Gender, n (male:female)	100:0	100:0
Age (years)	28.67 (9.03)	26.78 (8.21)
Education (years)	15.33 (2.35)	13.06 (2.75)
IQ-Full Scale	126.33 (11.56)	102.67 (14.32)
IQ-Verbal	126.95 (11.24)	105.83 (14.54)
IQ-Performance	119.67 (11.35)	99.11 (15.22)
Clinical Measures		
**MINI**[a]	0	9
Depression		3
Dysthymia		1
Panic Disorder		2
Social phobia		2
Generalized anxiety disorder		2
Obsessive-compulsive disorder		1
**DSM-5**[a]	0	4
Tic disorder		3
Specific Phobia		1
Trichotillomania (hair-pulling)		1
Medication Status	0	10
Antidepressants		9
Antipsychotics		6
Psychostimulants		1
Benzodiazepines		1
**ADOS** total	-	11.17 (3.09)
**ADOS social communication**	-	10.17 (3.13)
**ADOS repetitive behaviors**	-	1 (0.77)
**Self-reported measures**		
**AQ**	15.90 (4.28)	31.27 (6.48)
**RBS-R (adulthood period)**	1.80 (2.48)	32.69 (23.09)
**RBS-R (chilhood period)**	1.75 (2.77)	28.64 (23.62)

[a] Refers to past comorbidity. Some of the participants were experiencing symptoms related to the reported diagnostic entities at the time of the study, but these were subclinical and/or secondary to autism symptoms. Legend: ADOS,

Autism Diagnostic Observation Schedule; AQ, Autism Spectrum Quotient; MINI, Mini-International Neuropsychiatric Interview; IQ, intelligence quotient; SD, standard deviation; RBS-R, Repetitive Behaviors Scale – Revised.

Supplementary Description of the fMRI-adapted Stop-Signal Task used for this study

We asked participants to respond by pressing one of two buttons (left or right) of the response box. They were instructed that a) they should quickly press the button on go trials (75% of the total number of trials) and withhold the response on stop trials (25% of the total number of trials, randomly presented), b) both stopping on stop trials and responding fast on go trials were equally important, c) go trials would be aborted after a fixed period of time, d) the task was adaptive to their performance so that at the end of each run the number of successful and failed stops was approximately equal, and e) stopping would not be possible in some of the stop trials.

Participants completed 3 runs of 120 trials (a total of 360 trials: 270 go + 90 stop trials). Within each run, blocks of 20 trials were interleaved with baseline/response preparation periods of 25 seconds, corresponding to response preparation phases. We randomly jittered the mean Inter-trial Interval (ITI; the time between the end of the previous trial and the start of the current trial) between 750 and 2750 ms to optimize statistical efficiency. The trial began with a black dot (250 ms) followed by an arrow pointing to the right or left side on the screen (go signal). The arrow was displayed within the central 2.25 degrees of the visual field. In 25% of trials (the stop trials), the arrow in the go signal turned red (stop signal). The trials ended at the button press or after 1250 ms if the participant did not respond (Successful Stopping or Omission Go).

The stop signal delay (SSD; the delay between a go signal and a stop signal) started at 200 ms and was dynamically changed according to an adaptive staircase procedure, with a 50% performance criterion. If the participant stopped successfully on a stop trial, the SSD latency of the following stop trial increased by 50 ms (up to a maximum of 950 ms), making the task more difficult for the next trial; if the participant failed, the SSD latency decreased by 50 ms (up to a minimum of 100 ms). In this way, at the end of the task, successful stopping was always approximately 50%. Feedback about task performance (mean reaction time, number of Omission go trials, and % of Successful Stopping) appeared on the screen at the end of each run. Before entering the MRI scanner, participants performed a training session (240 trials) of the task.

Supplementary Description of the self-report questionnaires

The Autism Spectrum Quotient[1] is a 50-item self-assessment instrument for measuring the degree to which any adult of normal IQ has traits associated with the autistic spectrum. Scores of $\geq$32 indicate the need for further clinical evaluation. The Portuguese adaptation[2] of the instrument was performed by a group of experts with experience in autism. In the present study, we used the total Autism Spectrum Quotient score for purposes of sample characterization only.

The Repetitive Behavior Scale-Revised (RBS-R)[3] is a 43-item informant-based rating scale to comprehensively assess the occurrence and severity of a variety of restricted and repetitive behavors in autism. Family members, caregivers, or professionals who know the subjects well are asked to rate the severity of their behaviourbehaviors according to a four-point Likert scale, ranging from 0 (“behavior does not occur”) to 3 (“behavior occurs and is a severe problem”). The internal consistency of the original RBS-R and its translations is good.[4,5,6,7] In the present study, we used the preliminary Portuguese version, resulting from a process of translation-back translation performed by our group, including four psychiatrist experts in autism and a psychologist with 15 years of experience in psychometric evaluation. We applied the *RBS-R* to assess the global severity of RBs during the previous month and the childhood period (0-12 years).

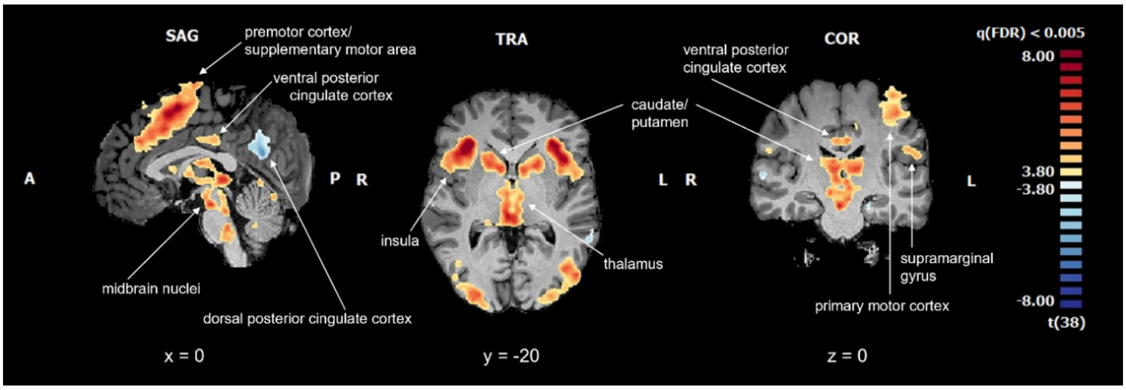

Supplementary Figure 1 Error-related whole-brain activations during the stop-signal task in individuals with autism and neurotypical subjects. The presented maps were extracted from the contrast between the failed stop and baseline/response preparation periods (*Failed Stop > Baseline/Response Preparation;* RFX, $t(38) = 3.80$, P-FDR = 0.005), including all the participants. Regions in yellow-orange tones represent positive changes in brain activity, and regions in blue tones represent negative changes in brain activity related to failed inhibitory responses. The brain map shows error-related activity changes in networks subserving response inhibition (striatum, thalamus, premotor, and primary motor cortex); instrumental (especially the dorsal regions of the striatum), value learning (midbrain nuclei, namely a large cluster including the ventral tegmental area and substantia nigra), and salience processing (insula and visual and visuomotor regions). There is also deactivation in the default mode network nodes (posterior cingulate cortex and medial temporal gyrus), which is consistent with the need to direct attention to external stimuli imposed by the task. *RFX*: random effects; *FDR*: false discovery rate. COR: coronal; SAG: sagittal: TRA: transversal.

Supplementary Table 2 List of significant clusters resulting from the whole-brain RFX analysis in individuals with autism and neurotypical subjects used the help define the relevant ROI for the main analysis

	MNI coordinates (peak)					
**Region**[a]	***x***	***y***	***z***	**Number of voxels**	***t***	***P***
Right supramarginal/ Angular gyrus	61	-44	32	14402	7.016	$< 1.00\times10^{-6}$
Bilateral FEF/ Premotor cortex/ SMA/ Broca opercularis/ Insula/ Basal ganglia/ Midbrain	-34	17	10	104888	10.182	$< 1.00\times10^{-6}$
Right Visual/ Visuoassociative cortex/ Fusiform gyrus/ Cerebellum	41	-68	-12	19407	8.950	$< 1.00\times10^{-6}$
Right cerebellum	29	-80	-35	1978	-6.533	$< 1.00\times10^{-6}$
Right dorsolateral PFC	36	44	34	4592	5.708	1.00×10^{-6}
Right visuomotor cortex	12	-74	36	1082	4.8	2.50×10^{-5}
Medial cerebellum medial	7	-73	-18	1544	5.654	2.00×10^{-6}
Pons	1	-35	-38	1278	6.399	$< 1.00\times10^{-6}$
Ventroposterior cingulate cortex	-4	-14	31	2404	6.484	$< 1.00\times10^{-6}$
Dorsoposterior cingulate cortex	-2	-62	19	4109	-7.569	$< 1.00\times10^{-6}$
Left primary motor cortex/ Supramarginal/ Angular gyrus	-47	-36	51	23359	6.296	$< 1.00\times10^{-6}$
Visual/ Visuoassociative cortex/ Fusiform gyrus/ Cerebellum L	-35	-53	-33	19579	7.192	$< 1.00\times10^{-6}$
Left anterior PFC	-32	49	19	407	4.599	4.60×10^{-5}
Left posterior angular gyrus	-45	-67	34	5367	-6.319	$< 1.00\times10^{-6}$
Left anterior angular gyrus	-50	-43	28	1247	5.9	1.00×10^{-6}
Left supramarginal gyrus	-57	-22	20	771	5.616	2.00×10^{-6}
Left medial temporal gyrus	-58	-47	-4	612	-5.405	4.00×10^{-6}

[a] Listed clusters were selected based on brain activity contrast between failed stop trials and baseline/response preparation (*Failed Stop* > *Baseline/Response Preparation*; RFX, $t(38) = 3.80$, P-FDR = 0.005. Minimum cluster threshold = 50 voxels). Legend: FEF, frontal eye fields; IFC, inferior frontal cortex; PCC, posterior cingulate cortex; PFC, prefrontal cortex; SMA, supplementary motor area.

Supplementary Table 3 Performance on the stop-signal task and between-group comparison, adjusted for IQ level

	**Neurotypical** (n = 21) *Mean* (*SD*)	**Autism** (n = 19) *Mean* (*SD*)	***Statistics***[a]	***df***	***P***
Omission Go	2.51 (3.34) %	1.22 (2.7) %	$Z = 1.037$	36	0.315
Go RT	694.09 (195.49) ms	613.28 (172.99) ms	$Z = 3.112$	36	0.086
SSD	428.72 (213.52) ms	370.55 (195.4) ms	$Z = 2.213$	36	0.146
SSRT	262.5 (48.04)	247.82 (39.49) ms	$Z = 0.680$	36	0.415
Successful Stopping	52.99 (4.84) %	51.95 (3.39) %	$Z = 0.765$	36	0.388

[a] The univariate effect of group on all task variables was non-significant. Legend: *df*, degrees of freedom; Go RT, mean reaction time on go trials; *SD*, standard deviation; SSD, stop signal delay; SSRT, stop signal reaction time.

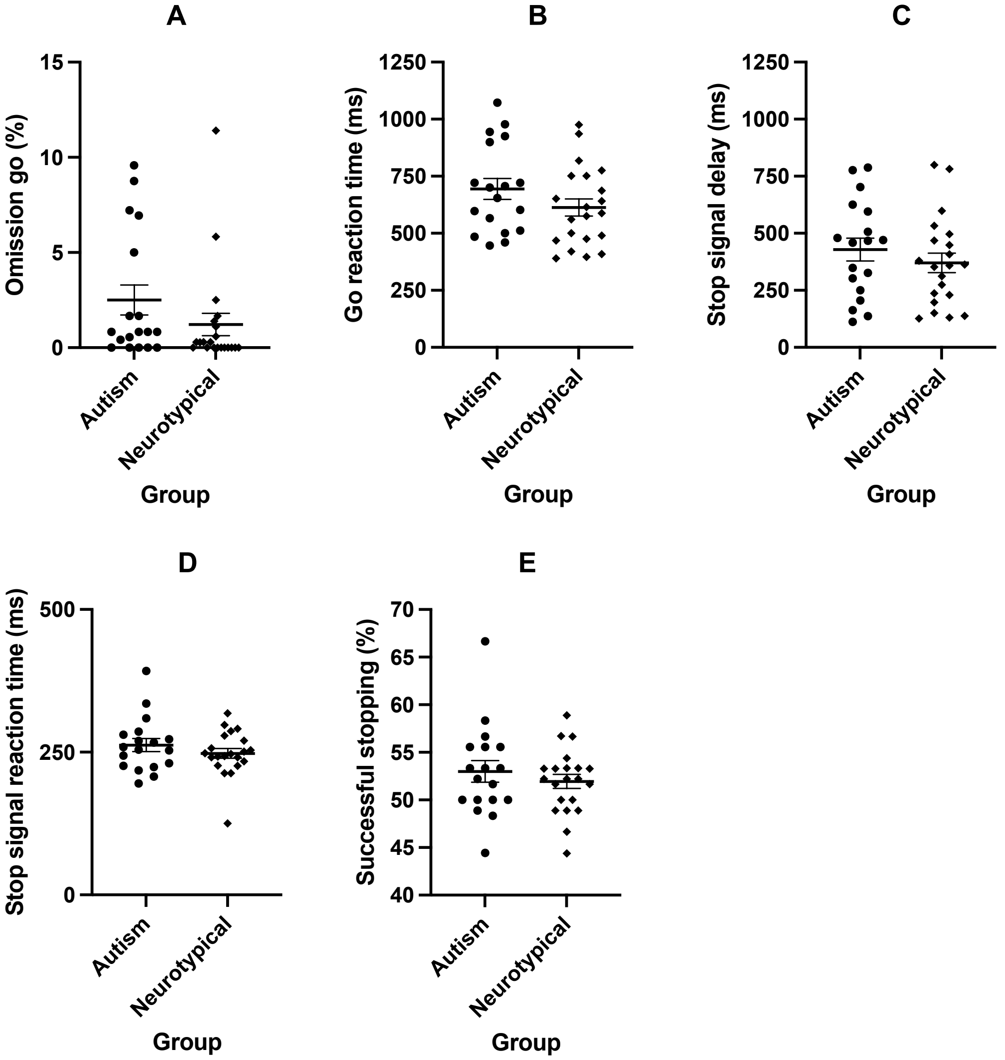

Supplementary Figure 2A-E Scatter plots of performance on the stop-signal task in individuals with autism (n = 18) and neurotypical subjects (n = 21). Task variables are represented in each figure. A: Proportion of omission

go trials, B: Mean reaction time on go trials, C: Stop-signal delay, D: Stop-signal reaction time, E: Proportion of successful stopping. Each data point represents an individual participant.

Supplementary Table 4.1 Three-factor repeated measures ANCOVA using IQ and antipsychotic medication use as covariates.

	df	*Mean square*	*F*	*P*[a]
Group	35	0.101	0.175	0.678
Inhibition phase	35	1.994	1.523	0.225
ROI	35	0.259	0.943	0.338
**Group x inhibition phase**	**35**	**11.999**	**9.165**	**0.005**
Group x ROI	35	0.377	1.373	0.249
Group x inhibition phase x ROI	35	0.001	0.002	0.964
WAIS	35	0.806	1.392	0.246
**Risperidone-equivalents**[b]	35	0.412	0.712	0.405

[a]Huynh-Feldt corrected.
[b]Antypsychotic dosages were converted to risperidone equivalents using an antipsychotic dose conversion calculator obtained in https://psychopharmacopeia.com/antipsychotic_conversion.php (last acessed in december 1, 2025).

Supplementary Table 4.2 *Post hoc* comparison of brain activation during phases of inhibition between autism and neurotypical groups

	**HC** (*n* = 21)	**Autism** (*n* = 18)	***t*-Test**[a]	***P***	**95% CI**	
					**Lower**	**Upper**
Striatum (failed inhibition)	*M = 1.3767* *SD = 0.908*	*M = 0.9061* *SD = 0.988*	*Z*(1, 36) = 1.557	0.220	-0.330	1.383
Midbrain (failed inhibition)	*M = 0.7150* *SD = 0.918*	*M = -0.014* *SD = 0.885*	*Z*(1, 36) = 5.218	**0.028**	**0.103**	**1.729**
Striatum (error processing)	*M = -0.0349* *SD = 0.636*	*M = 0.195* *SD = 1.123*	*Z*(1, 36) = 3.873	0.057	-1.513	0.023
Midbrain (error processing)	*M = -0.0575* *SD = 0.527*	*M = 0.290* *SD = 0.753*	*Z*(1, 36) = 3.714	0.062	-1.116	0.028

[a] IQ-covaried. Legend: *M*, mean; *SD*, standard deviation.

Supplementary Table 5 Partial (IQ-controlled) two-tailed correlations between brain activation and scores on the Repetitive Behaviours Scale – Revised in the autism and neurotypical groups.

**Brain activation**	**Repetitive behaviors**[a] *(n = 16)*		**Childhood repetitive behaviors**[a] *(n = 14)*	
	Autism	*Neurotypical*	*Autism*	*Neurotypical*
Striatum (failed inhibition)	*r* = 0.319 *P*-FDR = 0.329	*r* = -0.085 *P*-FDR = 0.729	*r* = 0.371 *P*-FDR = 0.329	*r* = 0.157 *P*-FDR = 0.520
Midbrain (failed inhibition)	*r* = 0.274 *P*-FDR = 0.369	*r* = -0.238 *P*-FDR = 0.326	*r* = -0.036 *P*-FDR = 0.908	*r* = -0.025 *P*-FDR = 0.919
Striatum (error processing)	***r* = -0.581** ***P*-FDR = 0.046**	*r* = -0.400 *P*-FDR = 0.089	***r* = -0.621** ***P*-FDR = 0.046**	*r* = -0.262 *P*-FDR = 0.279
Midbrain (error processing)	***r* = -0.639** ***P*-FDR = 0.04**	*r* = -0.090 *P*-FDR = 0.713	***r* = -0.747** ***P*-FDR = 0.024**	*r* = 0.039 *P*-FDR = 0.875

[a] *P*-values are FDR-corrected for the number of comparisons.

References:

1. Baron-Cohen S, Wheelwright S, Skinner R, Martin J, Clubley E. The Autism-Spectrum Quotient (AQ): Evidence from Asperger Syndrome/High-Functioning Autism, Males and Females, Scientists and Mathematicians. Vol 31.; 2001.

2. Castro SL, Lima CF. QA-Quociente de Espectro Autista, versão adultos (16+ anos). Adaptação portuguesa do The Autism Spectrum Quotient (AQ). 2010. Available at: https://docs.autismresearchcentre.com/tests/AQ_Portugues.pdf.. Accessed March 25, 2025.

3. Bodfish JW, Symons FJ, Parker DE, Lewis MH. Varieties of repetitive behavior in autism: Comparisons to mental retardation. Journal of autism and developmental disorders. 2000;30:237-243.

4. Martínez-González AE, Piqueras JA. Validation of the Repetitive Behavior Scale-Revised in Spanish-Speakers Participants with Autism Spectrum Disorder. J Autism Dev Disord. 2018;48(1):198-208. doi:10.1007/s10803-017-3276-0

5. Lam KSL, Aman MG. The repetitive behavior scale-revised: Independent validation in individuals with autism spectrum disorders. J Autism Dev Disord. 2007;37(5):855-866. doi:10.1007/s10803-006-0213-z

6. Inada N, Ito H, Yasunaga K, et al. Psychometric properties of the Repetitive Behavior Scale-Revised for individuals with autism spectrum disorder in Japan. Res Autism Spectr Disord. 2015;15-16:60-68. doi:10.1016/j.rasd.2015.01.002

7. Georgiades S, Papageorgiou V, Anagnostou E. Brief report: Repetitive behaviours in greek individuals with autism spectrum disorder. J Autism Dev Disord. 2010;40(7):903-906. doi:10.1007/s10803-009-0927-9
